# Prevalence of Malaria and Soil‐Transmitted Helminths and Associated Factors Among Pregnant Women Visiting Mizan‐Tepi University Teaching Hospital, Southwest Ethiopia

**DOI:** 10.1155/japr/2720965

**Published:** 2026-05-21

**Authors:** Tadesse Duguma, Surafel Fikadu, Sintayehu Kochito, Hayat Mohammed, Fikre Demango

**Affiliations:** ^1^ Department of Medical Laboratory Science, College of Medicine and Health Sciences, Mizan-Tepi University, Mizan-Aman, Ethiopia, mtu.edu.et

**Keywords:** associated factors, malaria, prevalence, soil-transmitted helminths, Southwest Ethiopia

## Abstract

**Background:**

The most common parasitic diseases in underdeveloped countries, particularly in sub‐Saharan Africa (SSA), are malaria and soil‐transmitted helminth (STH) infection, which primarily affect pregnant women and children under five.

**Objective:**

To determine the prevalence and associated factors of malaria and STH infections among pregnant women at Mizan‐Tepi University Teaching Hospital (MTUTH), Southwest Ethiopia, between March 1 and June 30, 2024.

**Methods:**

A cross‐sectional study was carried out among 330 pregnant women who attended an antenatal clinic at MTUTH between March 1 and June 30, 2024. Blood and stool samples were collected using a systematic random sampling procedure following the acquisition of signed informed consent. A standardized pretested questionnaire was utilized to gather clinical and sociodemographic information. Epi‐data and SPSS Version 27.0 were used for data entry and analysis. Bivariate and multivariable logistic regression analysis was done to assess the associated factors. Statistical significance was set at a *p* value of less than 0.05.

**Result:**

A prevalence of 25.2% (83/330) was recorded for malaria, and STH infections were identified among 29.4% (97/330) of pregnant women who participated, with *Ascaris lumbricoides* having the highest prevalence among the helminths. Residence (rural) and occupational status (farmer) showed statistically significant associations with malaria infection (AOR = 2.011; *p* = 0.024) and (AOR = 4.801; *p* = 0.048), respectively, whereas presence of stagnant water and use of insecticidal residual spray showed an association with malaria infection (AOR = 2.150; *p* = 0.008) and (AOR = 1.756; *p* = 0.047), respectively. Handwashing practice before meals and after toilet showed statistically significant association with STH infection (AOR = 6.468; *p* = 0.002) and (AOR = 8.826; *p* = 0.001), respectively.

**Conclusion:**

The high burden of malaria and STH among pregnant women highlights the need for integrated antenatal interventions, including early ANC attendance, health education, sanitation improvements, and preventive measures like insecticide‐treated nets and deworming, which would substantially lower these infections and improve maternal and fetal outcomes.

## 1. Introduction

Malaria and soil‐transmitted helminth (STH) infections are the most prevalent parasitic infections in developing countries, especially in sub‐Saharan Africa (SSA), affecting children under 5 years and pregnant women [1]. Globally in 2024, there were an estimated 610, 000 malaria deaths, an increase of 12,000 compared with 2023. Three countries, Madagascar, Ethiopia, and Yemen, accounted for 85% of the increase from 2023 to 2024 [2]. Heavy rainfall, stagnant waters, hot climate, and poor sanitation in the tropical and subtropical regions provide a good environment for the parasite to mature and continue breeding [3]. Pregnant women are more vulnerable to infections during pregnancy, contributed by a transient depression of cell‐mediated immunity that allows fetal allograft retention, and this interferes with resistance to infections [4]. Infection with intestinal helminths, particularly hookworms and malaria parasites, is associated with low hemoglobin levels, which leads to anemia during pregnancy [5].

### 1.1. Malaria Infection During Pregnancy

In 2024, 33 moderates to high transmission countries in the World Health Organization (WHO) African Region contain an estimated 36 million pregnancies, of which an estimated 13 million (36%) were infected with malaria [2]. Indeed, several studies conducted in Ghana, Nigeria, and Ethiopia noted most malaria infections among pregnant women were caused by *Plasmodium falciparum* [6–8]. According to the pooled estimates of 253 studies globally, the overall prevalence of malaria in pregnant women was recorded to be 18.95%, with the highest proportion observed in Africa, approximating 21.50% [9]. The residence of pregnant women, whether rural or urban, can also influence the prevalence of malaria infection. Findings of studies in Nigeria and Benin noted pregnant women who resided in urban areas were more prone to malaria infection than those in rural areas because of poor housing, drainage of mosquito breeding sites, and lack of access and use of insecticide‐treated net (ITN) and malaria chemoprophylaxis [10, 11].

Knowing risk factors associated with malaria infection will help pregnant women to observe effective measures to prevent and control malaria. A study conducted in Nigeria among pregnant women noted that those who had no formal education were more prone to malaria infection than those who had primary, secondary, and tertiary education since they did not know malaria preventive measures [11]. WHO recommends the use of ITN during pregnancy as one way to prevent infection with malaria. Sleeping under an ITN reduces human contact with mosquitoes, hence, reducing the chances of getting a malaria infection and associated mortality for mother and fetus. Findings of studies conducted in Ethiopia and Nigeria noted that pregnant women who were diagnosed with malaria infection did not use ITN [12, 13].

Intermittent preventive therapy (IPT) is given to pregnant women to prevent malaria during pregnancy and decrease the risk of maternal anemia [14]. The WHO recommends two dosages for malaria prophylaxis for every pregnant woman during their first and second trimesters to prevent malaria infection [15]. Studies from SSA noted that pregnant women who received malaria prophylaxis were less likely to have malaria infection [16–18].

Clearing bushes and draining stagnating water for the elimination of mosquitoes and for individual protection, including the use of insect‐treated mosquito nets, insect repellents, and indoor residual sprays (IRSs), are some of the methods used to prevent malaria. Health education strategies on prevention and the importance of control measures and community involvement in the creation and promotion of malaria awareness have shown a significant impact on the reduction of malaria incidence and prevalence [19].

### 1.2. STHs Infection During Pregnancy

STH infections are public health problems worldwide across the tropics and subtropics, especially in many developing countries. STHs are common in areas where there is a lack of safe water supply, poor personal hygiene, and poor sanitation [20]. The main STH species affecting pregnant women are the roundworms (*Ascaris lumbricoides*), whipworms (*Trichuris trichiura*), and hookworms [21].

Globally, more than 1.5 billion people have STH infections. *A*. *lumbricoides* affects approximately 1 billion people, whereas *T*. *trichiura* affects about 604 million people in SSA [22]. *A*. *lumbricoides* is the most prevalent STH among pregnant women. Indeed, findings from studies in western Kenya, Nigeria, and Ghana noted that *A*. *lumbricoides* was the most predominant parasite among the pregnant women [6, 23, 24]. Transmission of STH occurs when eggs passed in the feces of infected people contaminate the soil in areas with poor sanitation [25]. The eggs hatch into larvae and then mature into helminths. Mature helminths produce thousands of eggs in the intestines. Infection occurs after ingestion of eggs that are attached to raw vegetables (not washed or not peeled), contaminated water sources, and eggs ingested by eating contaminated soil and eating food without washing their hands. Severe infection can lead to pneumonia once the larva invades the lungs, leading to cough, bloody sputum, fever, and shortness of breath. In the intestine, it causes abdominal pains, constipation, diarrhea, and intestinal ulcers, which may lead to anemia [10].

STH infections during pregnancy lead to increased nutrient malabsorption rates, with *A*. *lumbricoides* competing for vitamin A in the intestines. Infected pregnant women usually have no appetite, hence, diminished nutritional intake and poor physical fitness. This can cause anemia in the mother, which leads to low birth weight, impaired milk production, and increased risk of mortality to the infant [26]. Hookworm infection affects about 44 million pregnant women globally [27]. Good sanitation in rural and urban areas, such as proper disposal of human waste and observing good personal hygiene, is a good measure to prevent infections. Findings from Kenya and Ethiopia noted that pregnant women from rural areas were more likely to be infected due to environmental sanitation, low socioeconomic status, and poor refuse disposal [28, 29].

Studies in Ethiopia and the Coastal and Northern Rift Valley in Kenya noted that pregnant women who washed their hands thoroughly with water and soap/detergent before meals and after visiting the toilet were not infected with intestinal helminths [30, 31].

The WHO recommended adequate sanitation and health education with the aim of reducing transmission and reinfection by encouraging healthy behaviors [32]. Preventive chemotherapy during pregnancy is aimed at reducing the infection intensity and protecting pregnant women from the morbidity associated with STH infections. The two anthelmintics recommended for the treatment of STH infections in pregnancy are albendazole (400 mg) and mebendazole (500 mg) since they are affordable and available in the health facilities, effective, easy to administer, and have fewer side effects [33]. The use of clean water, good sanitation, and wearing protective clothing and shoes reduces hookworm and schistosomiasis infection [34]. Our study area is also a malaria‐endemic zone with frequent malaria outbreaks. It has a tropical climate, which is characterized by hot and humid conditions and two rainy seasons. Heavy rainfall and stagnant waters, hot climate, and poor sanitation provide a good environment for malaria and helminth parasites to mature and continue breeding.

The objective of the study was to determine the prevalence of malaria and STH and associated factors among pregnant women attending antenatal care (ANC) at MTUTH. This will generate recommendations that would be useful in the design of interventions to help raise awareness among pregnant women in the prevention of malaria and intestinal helminths.

The findings of the study could help develop strategies for potential solutions for malaria and STH infection prevention and consequently improve pregnancy outcomes for mothers and infants, resulting in good health, well‐being, economic potential, and community development. The study could serve as baseline information for stakeholders to address vulnerable segments of the community and increase awareness on the prevention and control strategies. The study could also be used as a reference for any future research carried out in this area.

## 2. Materials and Methods

### 2.1. Study Design, Setting, and Period

A descriptive cross‐sectional hospital‐based study was conducted on pregnant women who were attending ANC at MTUTH from March 1 to June 30, 2024. The study was conducted at Mizan‐Tepi University Teaching Hospital, Mizan Aman, Southwest Ethiopia. Mizan‐Tepi University Teaching Hospital is a university hospital founded in 1986. Previously, the hospital was known by its name, “Mizan‐Teferi General Hospital,” during the Derg regime. And later Mizan Aman General Hospital until it was incorporated into Mizan‐Tepi University in 2016 and gained its current name, Mizan‐Tepi University Teaching Hospital. It is located in the southwestern part of Ethiopia, in a town called Mizan‐Teferi (recently named Mizan‐Aman Town). Mizan‐Aman Town is the capital town of Bench Sheko Zone (previously the capital of Bench Maji Zone), located 565 km southwest of Addis Ababa, the capital city of Ethiopia.

### 2.2. Study Participants

Pregnant women who visited the Mizan‐Tepi University Teaching Hospital from March 1 to June 30, 2024 served as the study cohort. All pregnant women in the age range of 15–49 years who visited the MTUTH during the study period and fulfilled the inclusion criteria were enrolled as the study participants.

### 2.3. Study Variables

Prevalence of malaria and STHs is the outcome variable, whereas the following were the independent variables of the study: sociodemographic and economic characteristics include age, marital status, residence, education level, employment status, and occupation.

Obstetric characteristics include trimester, gravidity status, and the number of ANC visits.

Behavioral habits include hand washing before meals and after visiting the toilet, the habit of biting nails, eating raw and unwashed fruits/vegetables, eating soil/rocks, sources of water for drinking, water preservation methods, use of ITNs, and IRS, whereas environmental factors include soil contact, sanitary facilities, and draining of stagnant water.

### 2.4. Study Population

All pregnant women attending ANC at MTUTH during the study period and fulfilling the inclusion criteria are enrolled as the study population.

### 2.5. Inclusion Criteria

Pregnant women who were attending the antenatal clinic during the study period and who provided informed consent are the inclusion criteria.

### 2.6. Exclusion Criteria

Pregnant women who did not give consent and who were on antimalarial and antihelminth medications are the exclusion criteria. Moreover, pregnant women with pre‐existing, chronic illnesses like HIV/AIDS, renal disease, and other medical conditions are also excluded.

### 2.7. Sample Size and Sampling Techniques

The sample size was calculated based on a single population proportion formula. Using this formula, the sample size was calculated as follows: by talking about the previous study conducted on the prevalence of soil‐transmitted malaria coinfection among pregnant women in Gilgel Gibe dam Area, Southwest Ethiopia (*p* = 7.7*%*) [35].

n=z2p 1−P¯    d2

where *n* = minimum sample size required, *Z* = *Z* scores corresponding to the level of confidence (95% = 1.96), *P* = the proportion in the target population estimated to have the characteristic being measured, *d* = margin of error = 3*%*, and *q* = 0.923.

n=1.962∗0.077∗10.077−0.032=303.36304≈

*n* = 304. After adding a 10% nonresponse rate, the sample size becomes 335.

#### 2.7.1. Sampling Technique

The systematic random sampling technique was employed to select pregnant women. Stool and blood samples were collected from each study participant to investigate the prevalence of malaria and helminth infections. By checking the ANC attendants′ logbook, we tried to assess the client flow for the past 2 months and found that 748 (*N*) pregnant women were attended to at the ANC clinic; accordingly, the sampling interval was calculated as (*K* = *N*/*n*); (748/335 = 2.23 ≈ 3). Participants were selected randomly, taking the first person through a lottery method to solve issues related to selection bias, and we proceeded to enroll every third pregnant woman (*k* = 3) who visited the hospital until we reached the desired sample size. The hospital′s daily patient flow is more than 350, which makes getting the required number of study participants for recruitment easy.

### 2.8. Data Collection and Laboratory Procedure

#### 2.8.1. Questionnaires on Sociodemographic and Clinical Data Collection Procedure

A well‐structured and pretested questionnaire, containing variables related to sociodemographic and behavioral habits, environmental conditions, and associated risk factor variables, was collected. The data were gathered through face‐to‐face interviews utilizing structured questionnaires adapted from previous studies conducted in Ethiopia.

#### 2.8.2. Stool Sample Collection and Laboratory Methods

All consenting pregnant women were provided with labeled screw‐capped stool containers, which were labeled with the code number of each participant. Clear instructions were given on how to collect the stool and transfer about 5 g of fresh stool sample into a container, and the saline wet mount was examined microscopically under 10× and 40× objectives.

#### 2.8.3. Malaria Parasite Examination and Parasite Identification

A capillary blood sample of 2‐ and 6‐*μ*L volume was collected from each pregnant woman to prepare thin and thick blood films for malaria parasite detection, respectively. The thin blood film was fixed by 70% methanol for 30 s and air‐dried. Both thin and thick films were stained using 10% Giemsa for 10 min. All asexual forms of malaria parasites (trophozoites and schizonts) in each preparation were microscopically identified using an oil immersion objective × 100 and recorded. At least 100 high‐powered fields were examined before the film was reported as negative.

#### 2.8.4. Quality Control

To assure the quality of data in the study, data collectors and supervisors were trained, and regular supervision and follow‐up were performed by supervisors and the principal investigator. Also, a regular check‐up for completeness and consistency of the data was done on a daily basis. A clinical and sociodemographic questionnaire was prepared in English, translated to Amharic and Benchigna, then back to English to ensure relevance and accuracy. Confidentiality of the data was kept, and the result will be disclosed by code. Two wet mount slides were prepared simultaneously for each participant to reduce the probability of false negative slides and compensate for the limited capability of a wet mount microscopy, and 10% buffered (pH 7.2) Giemsa stain was prepared for use every 24 h. Additionally, 5% randomly selected slides were reexamined by a senior laboratory technologist for confirmation. Moreover, the standard operating procedure (SOP) was strictly followed during the preanalytical, analytical, and postanalytical stages of laboratory investigations.

#### 2.8.5. Data Analysis and Interpretation

The data were coded, entered, checked by Epi‐data Version 4.6, and exported into SPSS Version 27.0 for analysis. Descriptive and inferential statistics were used to analyze the sociodemographic and obstetric characteristics, the prevalence of parasitic infections and their coinfection, and the behavioral and environmental characteristics associated with parasitic infections and their coinfections. Variables with a *p* ≤ 0.25 in the bivariate analysis were exported to multivariable logistic regression analysis. Bivariate and multivariable logistic regression were used to determine the association between malaria, intestinal helminth infection, and their co infection. A *p* value of less than 0.05 was considered statistically significant.

#### 2.8.6. Ethical Considerations

Ethical clearance with Approval Number CHS/00993/24 was obtained from the College of Health Science and Medicine at Mizan‐Tepi University. Before blood sample collection, the objective of the study and the procedure for sample collection were explained to the study participants. Written informed consent was obtained from the study participants. The information sheet and the purpose of the study were explained to each study participant. To ensure confidentiality of participants′ information, codes were used whereby the name of the participant and any identifier of participants was not to be written on the questionnaire. Voluntary participation clearly stated that they can choose to participate or not, and they can still receive all the services they usually do if they choose not to participate. Results of participants with parasitic infections, malaria, and/or intestinal helminths were sent immediately to the ANC department for treatment.

#### 2.8.7. Operational Definition of Terms

Operational definition of terms are as follows:

Malaria: It is a parasitic infection commonly transmitted by infected female Anopheles mosquitoes, which belong to the *Plasmodium* type.

Intestinal helminths: These are soil‐transmitted worms that can infect the gastrointestinal tract of humans.

Coinfection: simultaneous infection of a host organism by two or more pathogens.

Prevalence: It is the proportion of persons in a population who have a particular disease at a specific point in time or over a specific period.

Pregnant women: a woman having a developing embryo, fetus, or unborn offspring within the body.

## 3. Results

### 3.1. Socioeconomic and Demographic Characteristics

Data were collected from 330 pregnant women. The mean age of the study participants was 28.41 (SD = ±6.401), with 141 (42.7%) pregnant women aged between 25 and 29 years. Two hundred twenty‐five (68.2%) pregnant women were living in rural areas, whereas 105 (31.8%) pregnant women were living in urban areas. Two hundred thirty‐four (70.9%) pregnant women were married. One hundred fifty‐one (45.8%) pregnant women were illiterate. One hundred forty‐one (42.7%) pregnant women were housewives. One hundred eighty‐seven (56.7%) pregnant women were multigravida (Table 1).

**Table 1:** The sociodemographic and obstetric characteristics of pregnant women attending ANC clinic at MTUTH (*n* = 330).

Characteristics	Categories	Frequency (%)
Age (years)	15–19	7 (2.1)
	20–24	63 (19.1)
	25–29	141 (42.7)
	30–34	49 (14.8)
	35–39	58 (17.6)
	≥ 40	12 (3.6)

Residence	Urban	105 (31.8)
	Rural	225 (68.2)

Marital status	Single	60 (18.2)
	Married	234 (70.9)
	Separated	36 (10.9)

Educational status	Illiterate	151 (45.8)
	Primary	105 (31.8)
	Secondary	55 (16.7)
	College and above	19 (5.8)

Occupational status	Housewife	141 (42.7)
	Farmer	50 (15.2)
	Merchant	93 (28.2)
	Employed	46 (13.9)

Family monthly income (ETB)	Low (< 1000)	186 (56.4)
	Middle (1001–5000)	124 (37.6)
	High (> 5000)	20 (6.1)

Family size	< 5 members	95 (28.8)
	≥ 5 members	235 (71.2)

Trimester	First trimester	82 (24.8)
	Second trimester	141 (42.7)
	Third trimester	107 (32.4)

Gravidity status	Primigravida	143 (43.3)
	Multigravida	187 (56.7)

Abbreviation: ETB, Ethiopian birr.

### 3.2. Health‐Seeking Behavior of the Study Participants

One hundred eight pregnant women reported that they did not visit the hospital for ANC service before this study, whereas nearly 60% of them have visited the institution at least once. More than 90% of the pregnant women seek health services when they feel fever, whereas 73.9% of them seek health services when they feel abdominal discomfort (Table 2).

**Table 2:** Health‐seeking behavior of the study participants.

Variable (questions)	Category	Frequency (%)
Do you visit health facility for ANC service before?	Yes	222 (67.3)
	No	108 (32.7)

Number of health facility visit.	< 4 times	196 (59.4)
	4 times	105 (31.8)
	> 4 times	29 (8.8)

Seek health service when feels febrile.	Yes	306 (92.7)
	No	24 (7.3)

Seeks health service when feels abdominal discomfort.	Yes	244 (73.9)
	No	86 (26.1)

How you experience the following in the past.	Tiredness	116 (35.2)
	Shortness of breath	91 (27.6)
	Rapid heartbeats	60 (18.2)
	Blood in urine	24 (7.3)
	Dizziness	39 (11.8)

Previous history of malaria infection during pregnancy?	Yes	48 (14.5)
	No	282 (85.5)

Have you received intermittent preventive therapy for malaria?	Yes	36 (10.9)
	No	294 (89.1)

### 3.3. Prevalence of Malaria Infection

**Figure 1:**
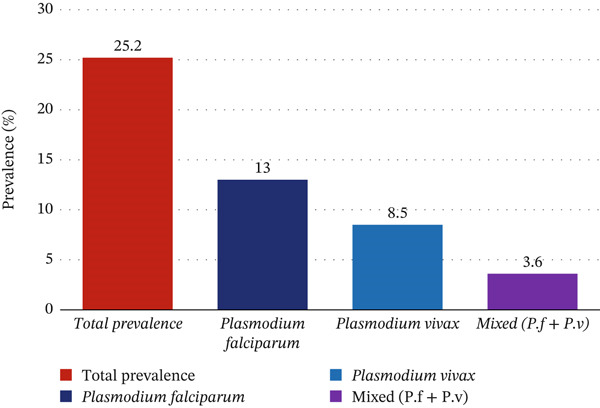
Malaria parasites examination and identification among pregnant women attending ANC clinic at MTUTH, Southwest Ethiopia.

Eighty‐three (25.2%) pregnant women had malaria infections, of which 43 (13.0%) were caused by *P*. *falciparum*, 28 (8.5%) by *Plasmodium vivax,* and 12 (3.6%) were mixed infections (Figure 1).

### 3.4. Behavioral Measures Used to Prevent Malaria Infection

**Figure 2:**
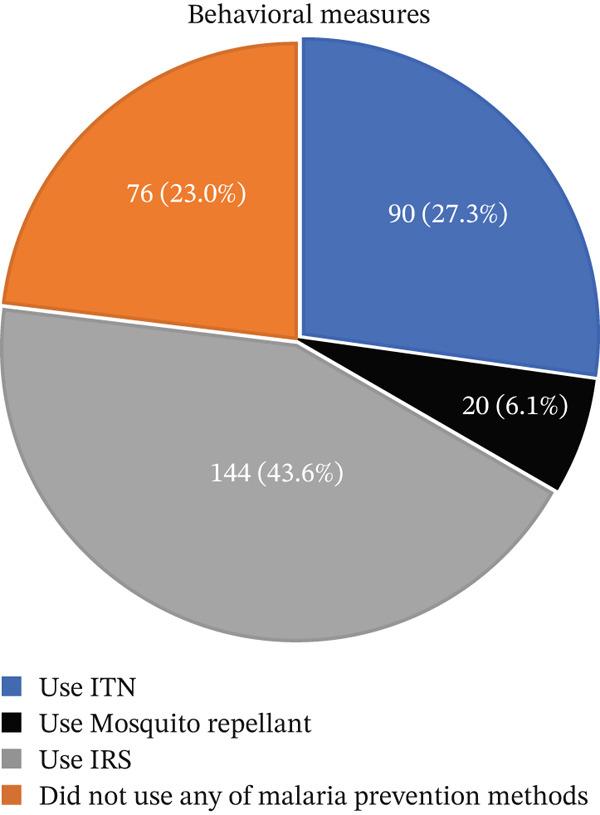
Behavioral measures used to prevent malaria infection by pregnant women attending ANC clinic at MTUTH.

Only 90 (27.3%) pregnant women used ITNs. Among pregnant women who did not use ITN, 31 (9.4%) pregnant women reported it was torn, and 24 (7.3%) pregnant women reported they could not find ITN on the market to buy. Sixty‐six (20.0%) of them reported that they did not own it. Seventy‐six (23.0%) of the study participants reported that they did not use any of the malaria prevention methods (Figure 2).

### 3.5. Prevalence of STH Infections

Ninety‐seven (29.4%) pregnant women had STH infection, of which 38 (11.5%) were infected with *A*. *lumbricoides*, 23 (7%) with *T*. *trichiura*, 19 (5.8%) with hookworm, 11 (3.3%) with *Strongyloides stercoralis*, and six (1.8%) with *Hymenolepis species.* Moreover, 16 cases of *Schistosoma mansoni were recorded* from the stool sample of the study participants.

### 3.6. Prevalence of Malaria and STH Infection

**Figure 3:**
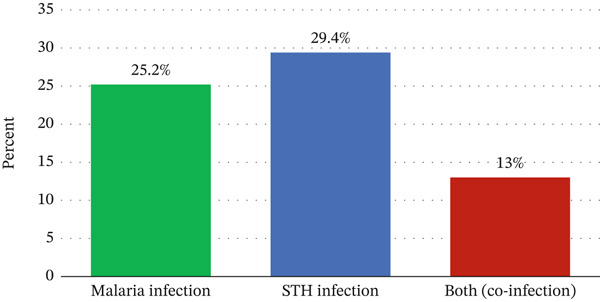
Prevalence of malaria, STH infection, and coinfection among pregnant women attending ANC clinic at MTUTH, Southwest Ethiopia.

Eighty‐three (25.2%) of pregnant women had *Plasmodium* infection, whereas 97 (29.4%) pregnant women were infected with at least one soil‐transmitted worm. Moreover, 43 (13.0%) pregnant women had both malaria and STH infection (coinfection) (Figure 3).

### 3.7. Sociodemographic and Factors Associated With Malaria Infection

Thirty (9.1%) pregnant women aged 25–29 years were found to have malaria infection, which is the highest among the age categories. The highest overall malaria infection was recorded in rural residents 65 (19.7%), followed by illiterate women 44 (13.3%). Moreover, housewives and those with low income take major shares from their respective categories, as 41 (12.4%) & 40 (12.1%). Residence and occupational status (farmers) showed statistically significant association with malaria infection among sociodemographic factors with a *p* value of *p* = 0.024 and *p* = 0.048, respectively. The presence of stagnant water and the use of IRS showed statistically significant associations with malaria infection with *p* = 0.047 and *p* = 0.008, respectively. Fifty‐eight (17.6%) of the pregnant women did not use ITN (Table 3).

**Table 3:** Sociodemographic and associated factors with malaria infection among pregnant women attending ANC clinic at MTUTH (*n* = 330).

Variables	Category	Malaria infection		Odds ratio (95% CI)			
		Yes *n* (%)	No *n* (%)	COR	*p*value	AOR	*p*value
Age (years)	15–19	2 (0.6)	5 (1.5)	Ref		Ref	
	20–24	21 (6.4)	42 (12.7)	0.833 (0.102–6.783)	0.865		
	25–29	30 (9.1)	111 (33.6)	0.667 (0.163–2.724)	0.572		
	30–34	12 (3.6)	37 (11.2)	1.233 (0.314–4.841)	0.764		
	35–40	15 (4.5)	43 (13.0)	1.028 (0.239–4.425)	0.971		
	> 40	3 (0.9)	9 (2.7)	0.956 (0.228–4.004)	0.950		

Residence	Urban	18 (5.5)	87 (26.4)	Ref		Ref	
	Rural	65 (19.7)	160 (48.5)	1.964 (1.095–3.520)	0.023	2.011 (1.097–3.688)	0.024 ^∗^

Education status	Illiterate	44 (13.3)	107 (32.4)	0.286 (0.063–1.291)		5.861 (0.621–55.298)	0.123
	Primary	25 (7.6)	80 (24.2)	0.376 (0.081–1.743)	0.211	3.873 (0.403–37.219)	0.241
	Secondary	12 (3.6)	43 (13.0	0.422 (0.085–2.086)	0.290	3.586 (0.340–37.810)	0.288
	College and above	2 (0.6)	17 (5.2)	Ref		Ref	

Occupational status	Housewife	41 (12.4)	100 (30.3)	0.366 (0.144–0.929)	0.034	3.252 (0.838–12.619)	0.088
	Farmer	17 (5.2)	33 (10.0)	0.291 (0.103–0.823)	0.020	4.801 (1.014–22.735)	0.048 ^∗^
	Merchant	19 (5.8)	74 (22.4)	0.584 (0.216–1.580)	0.290		
	Employed	6 (1.8)	40 (12.1)	Ref		Ref	

Average monthly family income (ETB)	Low (≤ 1000)	40 (12.1)	146 (44.2)	1.965 (0.735–5.253)	0.178	0.421 (0.097–1.828)	0.248
	Middle (1001–5000)	36 (10.9)	88 (26.7)	1.316 (0.486–3.568)	0.589		
	High (> 5000)	7 (2.1)	13 (3.9)	Ref		Ref	

Family size	≤ 5 members	22 (6.7)	73 (22.1)	Ref		Ref	
	> 5 members	61 (18.5)	174 (52.7)	1.163 (0.665–2.034)	0.596		

Use ITN	Yes	25 (7.6)	65 (19.7)	Ref		Ref	
	No	58 (17.6)	182 (55.2)	0.829 (0.479–1.433)	0.501		

Use of IRS	Yes	25 (7.6)	119 (36.1)	Ref		Ref	
	No	58 (17.6)	128 (38.8)	2.157 (1.268–3.669)	0.005	2.150 (1.222–3.783)	0.008 ^∗^

Use mosquito repellant	Yes	4 (1.2)	16 (4.8)	Ref		Ref	
	No	79 (23.9)	231 (70.0)	0.408 (0.107–1.557)	0.190	0.325 (0.078–1.352)	0.122

Presence of stagnant water near home	Yes	56 (17.0)	135 (40.9)	1.721 (1.020–2.903)	0.042	1.756 (1.006–3.065)	0.047 ^∗^
	No	27 (8.2)	112 (33.9)	Ref		Ref	

Abbreviations: AOR, adjusted odds ratio; CI, confidence interval; COR, crude odds ratio; IRS, insecticidal residual spray; ITN, insecticide‐treated net, Ref, reference category.

^∗^Indicate significant association.

### 3.8. Factors Associated With STH Infection

#### 3.8.1. Sociodemographic Factors Associated With STH Infection

Forty‐two (12.7%) pregnant women aged 25–29 years had STH infection. Fifty‐eight (17.6%) pregnant women who had STH infection were from rural areas. Thirty‐nine (11.8%) pregnant women who had STH infections were illiterate. Forty‐two (12.7%) pregnant women were housewives with STH infections. Across different sociodemographic factors that were studied, residence (rural) and family size (> 5) were found to be associated with STH infection (Table 4).

**Table 4:** Sociodemographic factors associated with STH infection among pregnant women attending ANC clinic at MTUTH (*n* = 330).

Variables	Category	STH infection		Odds ratio (95% CI)			
		Yes *n* (%)	No *n* (%)	COR	*p*value	AOR	*p*value
Age (years)	15–19	3 (0.9)	4 (1.2)	0.444 (0.061–3.242)	0.424		
	20–24	17 (5.2)	46 (13.9)	0.902 (0.218–3.732)	0.887		
	25–29	42 (12.7)	99 (30.0)	0.786 (0.203–3.048)	0.727		
	30–34	14 (4.2)	35 (10.6)	0.833 (0.196–3.539)	0.805		
	35–40	18 (5.5)	40 (12.1)	0.741 (0.179–3.065)	0.679		
	> 40	3 (0.9)	9 (2.7)	Ref		Ref	

Residence	Urban	39 (11.8)	66 (20.0)	Ref		Ref	
	Rural	58 (17.6)	167 (50.6)	0.588 (0.358 ‐ 0.965)	0.036	0.570 (0.379 ‐ 0.857)	0.007∗

Education status	Illiterate	39 (11.8)	112 (33.9)	0.727 (0.156–3.390)	0.685		
	Primary	31 (9.4)	74 (22.4)	0.833 (0.178–3.906)	0.817		
	Secondary	20 (6.1)	35 (10.6)	1.147 (0.225–5.840)	0.868		
	College and above	7 (2.1)	12 (3.6)	Ref		Ref	

Occupational status	Housewife	42 (12.7)	99 (30.0)	1.383 (0.454–4.208)	0.568		
	Farmer	17 (5.2)	33 (10.0)	1.585 (0.397–6.335)	0.514		
	Merchant	27 (8.2)	66 (20.0)	1.369 (0.424–4.416)	0.599	1.701 (0.637–4.538)	0.289
	Employed	11 (3.3)	35 (10.6)	Ref		Ref	

Average monthly income (ETB)	Low(< 1000)	51 (15.5)	135 (40.9)	0.545 (0.135–2.191)	0.392		
	Middle(ETB1001–ETB5000)	38 (11.5)	86 (26.1)	0.624 (0.149–2.619)	0.519		
	High(> 5000 ETB)	8 (2.4)	12 (3.6)	Ref		Ref	

Family size	≤ 5 members	35 (10.6)	60 (18.2)	Ref		Ref	
	> 5 members	62 (18.8)	173 (52.4)	1.628 (0.979–2.705)	0.060	0.593 (0.390 ‐ 0.901)	0.014∗

Abbreviations: AOR, adjusted odds ratio; CI, confidence interval, COR, crude odds ratio; Ref, reference.

^∗^Significant association.

#### 3.8.2. Risk Factors Associated With STH Infection

One hundred ninety‐seven (59.7%) pregnant women who washed their hands before meals had no STH infection, and also 193 (58.5%) pregnant women who washed their hands after visiting the toilet/latrine had no STH infection. Twenty‐four (7.3%) pregnant women who did have a habit of biting their nails were found to be infected with intestinal helminths. Across different risk factors that were studied to have possible association, both handwashing practice before meals and after toilet showed statistically strong association with STH infection with a *p* value of.002 and.001, respectively. Moreover, washing fruits before eating showed statistically significant associations with STH infection (*p* = 0.044) (Table 5).

**Table 5:** Risk factors associated with soil‐transmitted helminths infection among pregnant women attending ANC clinic at MTUTH (*n* = 330).

STH infection				Odds ratio (95% CI)			
Variables	Category	Yes *n* (%)	No *n* (%)	COR	*p*value	AOR	*p*value
Practice of handwashing habit before meal.	Regularly	36 (10.9)	197 (59.7)	Ref		Ref	
	Sometimes	61 (18.5)	36 (10.9)	0.108 (0.063–0.186)	< 0.001	6.468 (1.974–21.187)	0.002 ^∗^

Practice of handwashing after visiting toilet.	Regularly	25 (7.6)	193 (58.5)	Ref		Ref	
	Sometimes	72 (21.8)	40 (12.1)	0.072 (0.041–0.127)	< 0.001	8.826 (2.695–28.905)	0.00 ^∗^

Wash vegetables before eating?	Yes	11 (3.3)	57 (17.3)	Ref		Ref	
	No	86 (26.1)	176 (53.3)	0.395 (0.197–0.791)	0.009	1.336 (0.296–6.032)	0.706

Wash fruits before eating?	Always	23 (7.0)	146 (44.2)	5.011 (2.236–11.234)	< 0.001	0.131 (0.018–0.949)	0.044 ^∗^
	Sometimes	59 (17.9)	68 (20.6)	0.910 (0.425–1.949)	0.808	0.543 (0.079–3.716)	0.533
	Not at all	15 (4.5)	19 (5.8)	Ref		Ref	

Eating soil/rocks.	Yes	14 (4.2)	20 (6.1)	Ref		Ref	
	No	83 (25.2)	213 (64.5)	1.796 (0.867–3.722)	0.115	0.783 (0.130–4.717)	0.790

Habit of biting fingernails.	Yes	24 (7.3)	18 (5.5)	Ref		Ref	
	No	73 (22.1)	215 (65.2)	3.927 (2.017–7.646)	< 0.001	0.499 (0.105–2.371)	0.382
Practice of the shoe‐wearing habit?	Yes	55 (16.7)	201 (60.9)	4.797 (2.773–8.298)	< 0.001	0.242 (0.057–1.022)	0.054
	No	42 (12.7)	32 (9.7)	Ref		Ref	

Source of drinking water.	River	19 (5.8)	6 (1.8)	0.152 (0.050–0.466)	< 0.001	2.083 (0.124–35.055)	0.610
	Tap water	46 (13.9)	147 (44.5)	1.543 (0.752–3.165)	0.237	0.378 (0.058–2.484)	0.311
	Well	18 (5.5)	51 (15.5)	1.368 (0.594–3.149)	0.462		
	Stream	14 (4.2)	29 (8.8)	Ref		Ref	

Use of water preservation methods	Yes	24 (7.3)	78 (23.6)	Ref		Ref	
	No	73 (22.1)	155 (47.0)	0.653 (0.382–1.116)	0.119	2.056 (0.571–7.405)	0.270

Abbreviations: AOR, adjusted odds ratio; CI, confidence interval; COR, crude odds ratio; Ref, reference category.

^∗^Indicate significant association.

## 4. Discussion

Malaria during pregnancy continues to be a significant cause of illness for mothers, especially in areas where it is common. This study involved 335 pregnant women and achieved a high response rate of 98.5%, with only five participants opting not to provide a blood sample. The results showed a malaria prevalence of 25.2% among those studied.

When we compare this statistic with existing literature, we see a complex picture. This prevalence is lower than the 36% estimated by the WHO for malaria associated with pregnancy in Africa, which varies from 27.0% in East and Southern Africa to 40.1% in Central Africa [36]. However, it is much higher than the pooled prevalence from two global systematic reviews and meta‐analyses, which reported rates of 12.72% and 18.95% [9, 37], respectively. Conversely, our finding closely matches systematic reviews focused on SSA, which report pooled prevalences of 26.1% and 28.31% [38, 39]. This comparison highlights that malaria during pregnancy continues to pose a significant burden, aligning with regional estimates. The prevalence found in our study is similar to the 26.5% reported in Nigeria, but higher than the 21.6% and 16.5% seen in Western Kenya and Ghana, respectively [40–42]. These differences likely stem from local climate conditions, the effectiveness of vector control programs, and the use of preventive measures. Among those infected, *P*. *falciparum* was the most common species, which matches findings from studies in Ethiopia and Nigeria [11, 43, 44]. Although previous exposure to malaria may lead to some immunity, including against placental malaria, it does not negate the need for protective measures. The WHO advises the use of ITNs as a key method for preventing malaria during pregnancy [45]. By decreasing contact between humans and mosquitoes, ITNs reduce the likelihood of infection and decrease maternal and fetal mortality. In our study, most pregnant women reported using ITNs, which correlated with a lower risk of malaria infection. This protective effect is supported by research from Kenya and Nigeria, which found that women diagnosed with malaria were often those who did not use an ITN [11, 46, 47]. Among nonusers in our study, common reasons for not using an ITN included having a torn net, not receiving one during ANC, or personal dislike. These concerns are echoed in studies from Nigeria, where women reported excessive sweating due to poor ventilation. Overall, the findings emphasize the effectiveness of ITNs as an important tool for controlling malaria in pregnant women. Sociodemographic and environmental factors also significantly influenced the results. Multivariable analysis showed that women living in rural areas were 2.01 times more susceptible to malaria than urban residents, and those working as farmers were 4.8 times more likely to be at a higher risk of infection. Additionally, the lack of IRS and living close to standing water increased the odds of infection by 2.15 and 1.76 times, respectively.

The study also found that 29.4% of pregnant women were infected with at least one STH. Although this prevalence is high, it is lower than findings from Ethiopia′s Oromia region (30%), Tigray region (51.5%), and pooled estimates from South and Southeast Asia (43.15%) and East Africa (38.54%) [31, 48, 49, 28]. However, it is significantly higher than rates reported in Nigeria (12.0%) and it also exceeds the 27.32% pooled prevalence from a national meta‐analysis in Ethiopia [6, 50]. Another global meta‐analysis reported a prevalence below 30% among pregnant women [51]. These geographical differences are likely influenced by variances in local parasite transmission dynamics, sanitation infrastructure, and hygiene practices.

In our study, *A*. *lumbricoides* was the most common STH, aligning with studies in Western Kenya, Nigeria, and Ghana [23, 40, 41]. The ongoing issue of STH infections is often linked to poor sanitation and inadequate personal hygiene. Our analysis confirmed that the lack of key preventive practices greatly increased the risk of infection. Women who only occasionally washed their hands before meals were over 6.468 times more likely to have an STH infection, and those who only sometimes washed their hands after using the toilet were over 8.826 times more likely to be infected. On the flip side, protective behaviors were very effective; consuming fruits only after washing them was linked to much lower odds of infection. The strong connection between handwashing and reduced STH infection reflects findings from other studies [31, 52, 53, 29].

The coinfection of malaria and STH needs further attention. In this study, coinfection was most common between *P. falciparum* and *A. lumbricoides*, supporting findings from Nigeria and Ethiopia [6, 54] but another study from Ethiopia showed a high coinfection rate between *P. vivax* and *A. lumbricoides* [55]. This is clinically important since STH infections can worsen malaria complications. Malaria during pregnancy can lead to severe anemia, which affects about 29% of women with severe *P. falciparum* malaria [56]. Concurrent STH infections, especially hookworm and *A. lumbricoides*, contribute to anemia by causing nutrient malabsorption, competing for vitamin A, and reducing appetite, thus worsening maternal and fetal health [57]. This interaction highlights the need for integrated control approaches. In conclusion, this study reveals a significant burden of both malaria and STH infections among pregnant women in the area studied. Although the malaria prevalence aligns with regional estimates from SSA, the STH prevalence is notably high and closely tied to hygiene practices. The findings stress the need for reinforced, integrated efforts during ANC. These should ensure universal access to and use of ITNs and IRS for malaria prevention, along with targeted health education to promote effective STH preventive behaviors, including consistent handwashing, proper sanitation, and wearing footwear. Addressing the dual burden of these infections is essential for shaping targeted interventions and ultimately improving maternal and child health outcomes.

### 4.1. Study Limitation

This study has some limitations, of which the sole use of microscopy as a means of diagnosis is one. That is not compared with the more sophisticated diagnostic methods like PCR or rapid diagnostic testing, since it has a lower sensitivity, which may result in underestimation of the actual prevalence of helminths and malaria and could introduce bias into the study′s findings, limiting the generalizability of the results to settings with more sensitive diagnostic technologies. Lastly, we found wide confidence intervals for few of our variables that can be taken as a limitation of the study.

## 5. Conclusion and Recommendation

This study revealed a high prevalence of malaria and STH despite the preventive measures put in place by the government to reduce the infections in a community. Handwashing practice before meals and after using the toilet, and washing fruits before eating were found to have a statistically strong association with STH infections, whereas the presence of stagnant water near home and the use of IRS were found to have a significant statistical association with malaria infection. Improving the overall personal and environmental sanitation and increasing the ITN and IRS coverage in the community are important to protect pregnant women from further health complications.

**Nomenclature**
AIDS: acquired immunodeficiency syndrome
ANC: antenatal clinic
AOR: adjusted odds ratio
CI: confidence interval
COR: crude odds ratio
HIV: human immunodeficiency virus
ITN: insecticide‐treated net
IRS: insecticidal residual spray
MTUTH: Mizan‐Tepi University Teaching Hospital
NGO: nongovernmental organizations
PCR: polymerase chain reaction
SOP: standard operating procedure
SPSS: Statistical Package for Social Sciences
SSA: sub‐Saharan Africa
STH: soil‐transmitted helminth
WHO: World Health Organization

## Funding

No funding was received for this manuscript.

## Conflicts of Interest

The authors declare no conflicts of interest.

## Data Availability

The data that support the findings of this study are available from the corresponding author upon reasonable request.
